# *MC1R* genotype as a predictor of early-onset melanoma, compared with self-reported and physician-measured traditional risk factors: an Australian case-control-family study

**DOI:** 10.1186/1471-2407-13-406

**Published:** 2013-09-04

**Authors:** Anne E Cust, Chris Goumas, Kylie Vuong, John R Davies, Jennifer H Barrett, Elizabeth A Holland, Helen Schmid, Chantelle Agha-Hamilton, Bruce K Armstrong, Richard F Kefford, Joanne F Aitken, Graham G Giles, D Timothy Bishop, Julia A Newton-Bishop, John L Hopper, Graham J Mann, Mark A Jenkins

**Affiliations:** 1 Cancer Epidemiology and Services Research (CESR), Sydney School of Public Health, The University of Sydney, Sydney, NSW 2006, Australia; 2 Section of Epidemiology and Biostatistics, Leeds Institute of Molecular Medicine, University of Leeds, Leeds, UK; 3 Westmead Institute for Cancer Research, University of Sydney at Westmead Millennium Institute and Melanoma Institute Australia, Sydney, Australia; 4 Viertel Centre for Research in Cancer Control, Cancer Council Queensland, Spring Hill, Brisbane, Australia; 5 Centre for Molecular, Environmental, Genetic and Analytic (MEGA) Epidemiology, Melbourne School of Population Health, The University of Melbourne, Melbourne, Australia; 6 Cancer Epidemiology Centre, Cancer Council Victoria, Melbourne, Australia

**Keywords:** *MC1R*, Risk prediction, Accuracy, Melanoma, Sun exposure, Early-onset, Pigmentation, Nevi

## Abstract

**Background:**

Melanocortin-1 receptor (*MC1R*) gene variants are very common and are associated with melanoma risk, but their contribution to melanoma risk prediction compared with traditional risk factors is unknown. We aimed to 1) evaluate the separate and incremental contribution of *MC1R* genotype to prediction of early-onset melanoma, and compare this with the contributions of physician-measured and self-reported traditional risk factors, and 2) develop risk prediction models that include *MC1R*, and externally validate these models using an independent dataset from a genetically similar melanoma population.

**Methods:**

Using data from an Australian population-based, case-control-family study, we included 413 case and 263 control participants with sequenced *MC1R* genotype, clinical skin examination and detailed questionnaire. We used unconditional logistic regression to estimate predicted probabilities of melanoma. Results were externally validated using data from a similar study in England.

**Results:**

When added to a base multivariate model containing only demographic factors, *MC1R* genotype improved the area under the receiver operating characteristic curve (AUC) by 6% (from 0.67 to 0.73; *P* < 0.001) and improved the quartile classification by a net 26% of participants. In a more extensive multivariate model, the factors that contributed significantly to the AUC were *MC1R* genotype, number of nevi and previous non-melanoma skin cancer; the AUC was 0.78 (95% CI 0.75-0.82) for the model with self-reported nevi and 0.83 (95% CI 0.80-0.86) for the model with physician-counted nevi. Factors that did not further contribute were sun and sunbed exposure and pigmentation characteristics. Adding *MC1R* to a model containing pigmentation characteristics and other self-reported risk factors increased the AUC by 2.1% (*P* = 0.01) and improved the quartile classification by a net 10% (95% CI 1-18%, *P* = 0.03).

**Conclusions:**

Although *MC1R* genotype is strongly associated with skin and hair phenotype, it was a better predictor of early-onset melanoma than was pigmentation characteristics. Physician-measured nevi and previous non-melanoma skin cancer were also strong predictors. There might be modest benefit to measuring *MC1R* genotype for risk prediction even if information about traditional self-reported or clinically measured pigmentation characteristics and nevi is already available.

## Background

Melanoma is one of the most common cancers and a leading cause of cancer death in young adults of European origin [1,2]. People identified as being at high risk of melanoma could likely benefit from regular skin checks and interventions to improve sun-protection behaviours [3,4]. Phenotypic characteristics (e.g. hair, eye and skin colour, skin sensitivity to sunlight, number of nevi (moles)), family history, past sun exposure and past history of skin cancer are usually the basis for discriminating individual risk of melanoma [5-7]. However, given the decreasing costs and increasing use of genetic testing, it is becoming more feasible to incorporate genetic risk factors into clinical risk prediction tools. Low penetrant genetic variants for the melanocortin-1 receptor (*MC1R*) gene [8,9] are very common in populations of European origin [10,11] and some of these variants have been associated with a 1.5 to 4-fold increased risk of melanoma [12-15]. *MC1R* variants are associated with sun-sensitive phenotypes but the association with melanoma appears to be mediated also through non-pigmentary pathways [12,15]. To date, only three, preliminary, melanoma risk prediction models have included *MC1R* genotype [16-18]. No study has formally assessed the contribution of *MC1R* genotype to melanoma risk prediction compared with traditional factors.

The Australian Melanoma Family Study is a multi-centre, population-based, case-control-family study of early-onset melanoma (diagnosis before 40 years of age) that has comprehensive data for *MC1R* genotype and traditional risk factors including phenotype, UV (ultraviolet) radiation and clinically measured nevus counts [19]. Using data from this study, we aimed to 1) evaluate the separate and incremental contribution of *MC1R* genotype to prediction of early-onset melanoma, and compare this with the contributions of physician-measured and self-reported traditional risk factors, and 2) develop risk prediction models that include *MC1R*, and externally validate these models using an independent dataset from a genetically similar melanoma population [20].

## Methods

### Study sample

The Australian Melanoma Family Study design, recruitment, data collection and participant characteristics have been previously detailed [19]. Cases and controls were living in Brisbane, Sydney or Melbourne, which comprise about 50% of Australia’s population. Approval for the study was obtained from the ethics committees of The University of Sydney, The University of Melbourne, The University of Queensland, Cancer Council Victoria, Queensland Cancer Register and Cancer Council NSW. All participants provided written, informed consent.

### Case participants

Cases were identified from population-based state cancer registries, diagnosed between 1st July 2000 and 31st December 2002 at ages 18–39 years with incident, histopathologically-confirmed, first-primary invasive cutaneous melanoma. A total of 629 cases were recruited; participation was 54% of those eligible and 76% of those contactable.

### Control participants

Population controls were aged between 18 and 39 years at the time of approach and had no history of invasive or *in situ* melanoma. They were selected from the electoral roll (registration to vote is compulsory for Australian citizens aged 18 years and over) and were frequency-matched to cases by city, age and sex. A total of 240 population controls were recruited; participation was 23% of those apparently eligible and 42% of those contactable.

Eligible spouse or friend controls were a spouse, partner, or friend nominated by a case as a potential control participant. They were eligible if they were at least 18 years of age and had no history of invasive or *in situ* melanoma; there were no other age, sex or residency restrictions. A potential control was nominated by 59% of cases. A total of 295 spouse or friend controls were recruited; participation was 80% of those nominated. Population-controls and spouse or friend-controls were combined into one control group as done previously [15,19].

### Questionnaire data

Data were collected by telephone interview using a structured questionnaire, which included detailed questions on sun exposure, phenotype, residence history, demographic information, ancestry and diagnoses of cancer and non-melanoma skin cancers (basal cell carcinoma and squamous cell carcinoma) [19,21]. Participants also reported their skin colour and type, eye colour, natural hair colour at age 18 years, usual tanning and sunburn response to prolonged or repeated exposure to sunlight in summer, sunbed use, the number of nevi covering the body (described pictorially as none, few, some, many), freckling in childhood and adulthood, and nevus count on the back. Reported melanoma in relatives was verified where possible [19].

### Clinical skin examinations

All case and control participants were invited to attend clinical skin examinations, which were conducted at dermatology clinics in Brisbane, Sydney, and Melbourne by dermatology trainees trained on the study protocol. A clinical skin examination was completed by 73% of cases, 55% of population controls and 67% of spouse or friend controls.

Measurement of nevi was based on international guidelines [22]. Separate counts were made for melanocytic nevi of 2-5 mm and >5 mm, raised nevi of >2 mm, and clinically atypical nevi of >2 mm, on 30 body sites. The number of solar lentigines on the upper back was recorded by using a 6-level picture scale. Natural hair colour at age 18 and eye colour were recorded using wig hair swatches and eye photographs. Reflected skin colour, a correlate of melanin content [23], was recorded using a hand-held reflectance spectrophotometer with standard reflectance at 685 nm. The multi-wavelength data quantify colour using the Commission Internationale de l'Éclairage L* a* b* colour space parameters [24]. Inner arm L* values describe base skin colour, b* values describe tanning, and a* values describe erythema [23-25].

### *MC1R* genotyping and classification

Blood samples were requested from all participants and were obtained from 597 (95%) cases, 220 (92%) population controls, and 256 (87%) spouse/friend controls. The methods for *MC1R* genotyping and classification have been described in detail elsewhere [15]. Briefly, we sequenced *MC1R* and classified variants D84E, R142H, R151C, I155T, R160W, D294H as ‘R’ variants and all other variants excluding synonymous changes and non-coding changes as ‘r’ variants. R variants have been shown to be strongly associated with the presence of ‘red hair colour phenotype’ (red hair, fair skin, freckling, poor sun sensitivity), whereas r variants generally have a weaker association with red-hair colour phenotype [12]. The association of the individual *MC1R* variants with melanoma risk in this sample has been described previously [15].

When *MC1R* genotype was added to the statistical models, it was added together as a group of seven separate variables: one for each of the six ‘R’ variants D84E, R142H, R151C, I155T, R160W, D294H, and one variable for all ‘r’ variants combined. Each of these variables was formatted to indicate the number of variant alleles (i.e. 0,1,2).

### Statistical analysis

In order to compare the contribution of *MC1R* genotype with all self-reported and clinical traditional risk factors simultaneously, we restricted this analysis to case and control participants who had: complete questionnaire data for the main risk factors examined, a clinical skin examination, *MC1R* genotype, self-reported exclusive European ancestry, and were aged < 45 years at interview. After exclusions, 676 participants remained for the analysis: 413 cases and 263 controls (115 population-controls and 148 spouse or friend controls). Data were analysed using SAS version 9.2 (SAS Institute, Cary NC) and statistical significance was inferred at two-sided *P* < 0.05.

### Model development

In the ‘base’ model, melanoma status was the outcome variable and covariates included demographic factors: age (quadratic), sex, city of recruitment (Brisbane, Sydney, Melbourne), and self-reported European ancestry (British/northern, southern, eastern/mixed/other European) to account for any difference in *MC1R* allele frequencies across ethnic groups [11].

We added *MC1R* genotype and traditional risk factors separately to the base model to evaluate their individual contribution to risk prediction. We also added the risk factors incrementally to the base model in order of their contribution to the area under the receiver operating characteristic (ROC) curve (AUC). To examine the contribution of traditional pigmentation characteristics, we created a pigmentation-related propensity-to-melanoma score (‘pigmentation score’) continuous variable that summarizes the contribution of six correlated, categorical phenotypic variables, including self-reported ability to tan, propensity to sunburn, childhood freckling, skin colour, eye colour, and hair colour [15]. For the more objectively-measured pigmentation score, the last three self-reported variables were replaced with physician-measured skin reflectance, eye colour, and hair colour.

Other self-reported variables that were tested in the models were number of nevi fitted as a categorical variable (none, few, some, many), previous diagnosis of non-melanoma skin cancer (yes, no), and ultraviolet (UV) radiation related exposures: total childhood sun exposure hours (quartiles), childhood blistering sunburns (none, ≤ 8, > 8) and lifetime sunbed use (none, 1–10, >10 sessions). The two childhood measures were chosen over other sun exposure measures such as lifetime, adulthood, weekday and holiday sun exposure, because they were more predictive of melanoma in our study sample. Other physician-measured variables that were tested in the models were separate nevus counts (≥ 2 mm, 2–5 mm, ≥ 5 mm, dysplastic, raised) and solar lentigines. We also included confirmed family history of melanoma in a first-degree relative.

### Measures of model performance

As measures of discrimination, i.e. the ability of a model to discriminate those who will develop melanoma from those who will not, we calculated: the AUC, which is equivalent to the concordance (*c*) statistic; the net reclassification improvement (NRI); discrimination slope; and the integrated discrimination index (IDI) [26-29]. To assess calibration, i.e. the agreement between observed and predicted outcomes, we used the Hosmer-Lemeshow goodness-of-fit test [26,30]. These measures were based on predicted probabilities of melanoma from the unconditional logistic regression models described above.

The AUC is equal to the probability that, for one case and one control chosen at random from the data set, the predicted probability of melanoma is higher for the case than for the control, and ranges from 0.5 (equivalent to a coin toss) to 1.0 (perfect discrimination). The NRI quantifies overall improvement in model sensitivity and specificity. A net improvement in risk classification implies upward reclassification of case participants and downward reclassification of control participants. The NRI was calculated by first fitting a ‘base model’ which grouped participants into quartiles of their predicted probability of melanoma; these quartile distributions were then compared to the ‘comparison model’. Improvement in sensitivity represents net reclassification of more cases into higher quartiles, improvement in specificity represents net reclassification of controls into lower quartiles, and overall improvement in classification combines the improvements in sensitivity and specificity. In the absence of clinically meaningful cut-points, we used quartiles to define risk categories as done elsewhere [27]. We also calculated the ‘category-free’ NRI, for which the definition of upward or downward movement is simplified to indicate any increase or decrease in probabilities of the outcome [31]. Discrimination slope was calculated as the difference between the mean predicted probability for cases and controls, and the IDI was calculated as the difference between discrimination slopes between the base and comparison models; both of these measures do not require predefined risk categories. As measures of overall model performance, we estimated the Brier score and Nagelkerke’s R^2^, which are measures of how well future outcomes are likely to be predicted by the model [30]; a higher Nagelkerke’s R^2^ and a lower Brier score indicates better predictability of the model.

As a measure of internal validation, we used 100 bootstrap samples to estimate the AUC and Nagelkerke’s R^2^ for the final models. Odds ratios (OR) for melanoma and their 95% confidence intervals were estimated using unconditional logistic regression models.

### External validation

We performed external validation of the final regression models using a population-based case–control study of melanoma from a geographically defined area of Yorkshire and the Northern region of the United Kingdom [32]. Case participants had incident pathologically confirmed invasive melanoma diagnosed between September 2000 and December 2005 (67% case participation). Control participants were identified from the cases’ family doctors (55% response) and were frequency-matched to cases by age and sex. A total of 841 case participants and 452 control participants, aged between 18 and 76 years, were included in this analysis. This study was conducted in tandem with the Australian case–control study and used a common protocol for collection of phenotype and sun exposure measures to facilitate comparisons among the datasets. We handled the data variables and analysis in the same way for both datasets.

## Results

### Characteristics of the study sample

Demographic characteristics and selected risk factors of early-onset cases and controls are shown in Table 1. Fifty-eight percent of cases and 40% of controls had at least one R allele. A previous non-melanoma skin cancer was reported by 8% of cases and 2% of controls.

**Table 1:** Demographic characteristics and selected risk factors for cases and controls

**Characteristic**	**Cases (n = 413)**	**Controls (n = 263)**
Male (%)	36	42
Female (%)	64	58
Median age in years^1^ (median, IQR)	33 (28–37)	35 (31–39)
European ancestry (%)		
British or northern European	75	60
Southern European	4	7
Other European or unknown	21	33
*MC1R* (%)		
Wild-type consensus alleles only	15	29
r only alleles	27	32
Any R allele	58	40
Number of nevi ≥ 2 mm (median, IQR)	205 (108–320)	67 (28–158)
Number of dysplastic nevi (%)		
0	55	76
1	11	11
2+	34	13
Previous non-melanoma skin cancer (%)	8	2
Confirmed family history of melanoma (%)	10	5
Pigmentation score, self-reported (%)		
1st quartile (lower risk)	8	24
2nd quartile	26	26
3rd quartile	24	25
4th quartile (higher risk)	42	25
Number of childhood blistering sunburns (%)		
0	65	69
≤ 8	17	19
> 8	18	12
Number of lifetime sunbed sessions (%)		
0	76	80
1–10	14	14
> 10	10	6

*IQR* interquartile range.

^1^ Age at diagnosis for cases and age at interview for controls.

### Separate contribution of *MC1R* genotype and traditional factors

Compared to the base model, the separate addition of *MC1R*, pigmentation score, nevi, non-melanoma skin cancer and solar lentigines each considerably improved the discriminative ability of the model, whereas inclusion of self-reported sun and sunbed exposure variables (childhood sun exposure, childhood blistering sunburns and lifetime sunbed use) resulted in minimal improvement, and inclusion of family history resulted in no improvement (Table 2).

**Table 2:** **Separate contributions of *MC1R *genotype, and self-reported and physician-measured traditional factors to risk prediction of melanoma, measured using the area under the receiver operating characteristic curve (AUC) and net reclassification improvement (NRI)**

**Risk factor**^**1**^	**AUC (95% CI)**	**Change in AUC from base model**^**2**^	**P**^**3**^	**Improvement in sensitivity**^**4**^		**Improvement in specificity**^**4**^		**Overall improvement in classification**^**4**^	
				**NRI (95% CI)**	**P**^**5**^	**NRI (95% CI)**	**P**^**5**^	**NRI (95% CI)**	**P**^**5**^
Base model with demographic^6^ factors only	0.67 (0.63, 0.72)								
*MC1R* all variants^7^	0.73 (0.69, 0.77)	0.058	<0.001	0.12 (0.05, 0.19)	0.001	0.14 (0.06, 0.23)	<0.001	0.26 (0.15, 0.37)	<0.001
‘R’ variants only	0.72 (0.68, 0.75)	0.041	0.001	0.04 (−0.03, 0.11)	0.25	0.17 (0.09, 0.25)	<0.001	0.21 (0.10, 0.31)	<0.001
‘r’ variants only	0.68 (0.64, 0.72)	0.004	0.48	0.03 (−0.02, 0.07)	0.28	0.02 (−0.04, 0.08)	0.52	0.05 (−0.03, 0.12)	0.24
*Self-reported risk factors*									
Nevi (none, few, some, many)	0.72 (0.68, 0.76)	0.048	0.001	0.15 (0.07, 0.23)	<0.001	0.09 (−0.00, 0.18)	0.06	0.24 (0.12, 0.36)	<0.001
Pigmentation score^8^	0.73 (0.69, 0.77)	0.053	<0.001	0.09 (0.03, 0.16)	0.004	0.12 (0.04, 0.20)	0.003	0.22 (0.11, 0.32)	<0.001
Sun & sunbed exposure^9^	0.69 (0.65, 0.73)	0.015	0.06	0.04 (−0.01, 0.09)	0.1	0.04 (−0.02, 0.10)	0.2	0.08 (0.00, 0.16)	0.04
Family history^10^	0.68 (0.64, 0.72)	0.006	0.4	0.01 (−0.02, 0.04)	0.6	0.03 (−0.01, 0.07)	0.2	0.04 (−0.01, 0.09)	0.2
Non-melanoma skin cancer^11^	0.70 (0.66, 0.74)	0.024	0.003	−0.03 (−0.06, 0.01)	0.2	0.09 (0.05, 0.13)	<0.001	0.06 (0.01, 0.12)	0.02
*Physician-measured risk factors*									
Nevi 2+ mm	0.79 (0.75, 0.82)	0.111	<0.001	0.10 (0.03, 0.17)	0.008	0.29 (0.20, 0.38)	<0.001	0.39 (0.28, 0.51)	<0.001
Nevi 2–5 mm	0.78 (0.75, 0.82)	0.108	<0.001	0.10 (0.03, 0.17)	0.006	0.30 (0.21, 0.39)	<0.001	0.40 (0.29, 0.52)	<0.001
Nevi 5+ mm	0.76 (0.72, 0.79)	0.082	<0.001	−0.01 (−0.08, 0.06)	0.8	0.33 (0.25, 0.42)	<0.001	0.32 (0.21, 0.44)	<0.001
Nevi dysplastic	0.70 (0.66, 0.74)	0.027	0.01	−0.04 (−0.10, 0.01)	0.1	0.15 (0.09, 0.21)	<0.001	0.10 (0.02, 0.19)	0.02
Nevi raised	0.74 (0.70, 0.77)	0.061	<0.001	−0.04 (−0.11, 0.03)	0.2	0.29 (0.21, 0.36)	<0.001	0.24 (0.14, 0.35)	<0.001
Pigmentation score^12^	0.72 (0.68, 0.76)	0.047	0.001	0.11 (0.04, 0.17)	0.001	0.09 (0.01, 0.17)	0.03	0.20 (0.09, 0.30)	<0.001
Solar lentigines	0.74 (0.70, 0.78)	0.063	<0.001	0.09 (0.02, 0.16)	0.01	0.17 (0.08, 0.26)	<0.001	0.26 (0.15, 0.37)	<0.001

*AUC* Area under the receiver operating characteristic curve, *NRI* Net reclassification improvement.

^1^ Each risk factor was separately added to the ‘base model’ to evaluate its influence on risk prediction.

^2^ The change in the AUC between the base model and the model with the additional risk factor included.

^3^ Chi-square p-value for the difference in the AUC when compared to the base model.

^4^ Based on quartile cut-points. Improvement in sensitivity is calculated from reclassification of cases, improvement in specificity is calculated from reclassification of controls, and the overall improvement in classification combines the improvements in sensitivity and specificity.

^5^ The p-value, representing the statistical significance of the NRI, was calculated using the methods of Pencina *et al.*[29].

^6^ Demographic factors include age, sex, city of recruitment and European ancestry.

^7^*MC1R* is included as separate continuous variables for each of the six ‘R’ variants and one combined ‘r’ variant variable.

^8^ Self-reported ‘pigmentation score’ is a continuous variable derived from several self-reported variables including: ability to tan, propensity to sunburn, skin colour, eye colour, hair colour and childhood freckling.

^9^ ‘Sun & sunbed exposure’ includes total childhood sun exposure hours (quartiles), childhood blistering sunburns (none, ≤ 8, > 8), and lifetime number of sunbed sessions (0, 1–10, >10).

^10^ Confirmed family history of melanoma in a first degree relative (yes/no).

^11^ A self-reported previous diagnosis of non-melanoma skin cancer.

^12^ Objectively-measured ‘pigmentation score’ is a continuous variable derived from objectively-measured: hair colour, eye colour and skin reflectance (inner arm b* measure), and self-reported: ability to tan, propensity to sunburn and childhood freckling.

When added to the base model, *MC1R* improved the AUC by 6%, sensitivity by 12% (95% CI 5-19%), specificity by 14% (95% CI 6-23%), and improved the quartile classification for a net 26% (95% CI 15-37%) of participants. Further examination showed that the six ‘R’ variants were responsible for most of the improvement to risk prediction, as together they increased the AUC by 4% (*P* = 0.001) and improved the quartile classification by a net 21% (95% CI 10-31%) of participants whereas the combined ‘r’ variants increased the AUC by less than 1% (*P* = 0.5) and the net reclassification improvement by 5% (−3-12%) (Table 2).

The contribution of traditional pigmentation characteristics to model improvement was similar for self-reported and the more objectively-measured pigmentation score. Physician-counted number of nevi ≥ 2 mm and 2–5 mm were the nevi variables most predictive of melanoma risk, whereas self-reported number of nevi had a more modest impact. There was no material change to any of our results in this paper when we repeated the models, replacing the single composite ‘pigmentation score’ variable with the six separate variables that comprise the pigmentation score (data not shown). We also tested hair colour as a separate variable and found that it contributed about half as much to the AUC compared to the pigmentation score variable.

### Incremental contribution of *MC1R* genotype and traditional factors

In a more extensive multivariate model where each risk factor was added incrementally to the base model in order of their contribution to increasing the AUC, only *MC1R*, number of nevi and history of non-melanoma skin cancer significantly improved the AUC for both the self-reported and physician-measured models (Table 3). Self-reported pigmentation score weakly increased (by 1%; *P* = 0.07) the AUC for the self-reported model already containing *MC1R*, nevi and non-melanoma skin cancer, whereas more objectively-measured pigmentation score did not increase the discrimination of the corresponding physician-measured model. *MC1R* and number of nevi were the only variables that produced significant quartile reclassification of cases and controls. Measures of sun and sunbed exposure and solar lentigines did not increase the discrimination of the models already containing the other factors; nor did number of dysplastic nevi or raised nevi, once number of nevi ≥ 2 mm (the most predictive nevus variable) was included in the physician-measured model.

**Table 3:** **Forward stepwise models showing the incremental contribution of *MC1R *genotype and traditional risk factors to risk prediction models for melanoma, with each factor added in order of their contribution to improving the AUC, shown separately for models using self-reported factors and physician-measured factors**

**Predictors in order of entry**^**1**^	**AUC (95% CI)**	**Incremental change in AUC**^**2**^	**P**^**3**^	**Incremental improvement in sensitivity**^**4**^		**Incremental improvement in specificity**^**4**^		**Overall incremental improvement in classification**^**4**^	
				**NRI (95% CI)**	**P**	**NRI (95% CI)**	**P**	**NRI (95% CI)**	**P**
Base model	0.67 (0.63, 0.72)								
*Self-reported model: including only self-reported nevus and phenotype risk factors*									
*MC1R* all variants	0.73 (0.69, 0.77)	0.058	<0.001	0.12 (0.05, 0.19)	0.001	0.14 (0.06, 0.23)	<0.001	0.26 (0.15, 0.37)	<0.001
Nevi (none, few, some many)	0.77 (0.73, 0.81)	0.038	0.001	0.14 (0.07, 0.20)	<0.001	−0.00 (−0.09, 0.08)	0.93	0.13 (0.02, 0.24)	0.02
Non-melanoma skin cancer	0.78 (0.75, 0.82)	0.012	0.02	−0.02 (−0.06, 0.01)	0.2	0.05 (0.01, 0.08)	0.01	0.02 (−0.03, 0.07)	0.4
Pigmentation score	0.79 (0.76, 0.83)	0.009	0.07	0.04 (−0.01, 0.08)	0.1	0.02 (−0.04, 0.08)	0.5	0.06 (−0.02, 0.13)	0.1
Sun & sunbed exposure	0.80 (0.76, 0.83)	0.004	0.4	0.02 (−0.02, 0.06)	0.4	0.03 (−0.02, 0.09)	0.2	0.05 (−0.01, 0.12)	0.1
Family history	0.80 (0.76, 0.83)	0.001	0.7	0.00 (−0.02, 0.03)	0.9	0.00 (−0.02, 0.03)	0.8	0.01 (−0.03, 0.04)	0.7
*Physician-measured model: including physician-measured nevus and phenotype risk factors where available*									
Nevi 2+ mm^5^	0.79 (0.75, 0.82)	0.111	<0.001	0.10 (0.03, 0.17)	0.008	0.29 (0.20, 0.38)	<0.001	0.39 (0.28, 0.51)	<0.001
*MC1R* all variants	0.82 (0.78, 0.85)	0.031	0.002	0.05 (−0.01, 0.10)	0.1	0.09 (0.02, 0.15)	0.007	0.13 (0.05, 0.22)	0.002
Non-melanoma skin cancer	0.83 (0.80, 0.86)	0.010	0.02	−0.01 (−0.04, 0.02)	0.4	0.03 (0.01, 0.06)	0.02	0.02 (−0.02, 0.06)	0.3
Pigmentation score^5^	0.83 (0.80, 0.86)	0.003	0.5	0.01 (−0.03, 0.05)	0.6	−0.03 (−0.08, 0.02)	0.2	−0.02 (−0.08, 0.04)	0.5
Solar lentigines^5^	0.83 (0.80, 0.86)	0.004	0.3	0.03 (−0.01, 0.07)	0.10	−0.01 (−0.05, 0.03)	0.7	0.02 (−0.03, 0.08)	0.4
Family history	0.83 (0.80, 0.86)	<0.001	1.0	0.00 (−0.01, 0.02)	0.5	−0.01 (−0.03, 0.01)	0.5	−0.00 (−0.03, 0.02)	0.8

*AUC* Area under the receiver operating characteristic curve, *NRI* Net reclassification improvement.

^1^ Each risk factor was added in a forward stepwise manner in order of their contribution to improving the AUC. The base model and individual risk factors are described in Table 2 footnotes.

^2^ The change in the AUC from the previous (incremental) model.

^3^ Chi-square p-value for the difference in the AUC when compared to the previous (incremental) model.

^4^ Based on quartile cut-points. Improvement in sensitivity is calculated from reclassification of cases, improvement in specificity is calculated from reclassification of controls, and the overall improvement in classification combines the improvements in sensitivity and specificity.

^5^ Objectively-measured risk factor – see Table 2 footnotes for descriptions.

### Selection and validation of final models, and measures of model performance

Based on improvement to the AUC, the final selected models for both the self-reported and physician-measured models included *MC1R*, nevi and non-melanoma skin cancer, in addition to demographic factors. Details of the models’ performance and validation are shown in Table 4. The AUC was higher for the physician-measured model (0.83, 95% CI 0.80-0.86) than for the self-reported model (0.78, 95% CI 0.75-0.82), a difference in the AUC of 0.043 (*P* < 0.001), reflecting better predictive ability of clinically-measured number of nevi than self-reported nevi.

**Table 4:** **Performance measures for the final selected self-reported and physician-measured risk prediction models for melanoma that include *MC1R*, nevi and non-melanoma skin cancer**

**Performance measure**	**Base model**^**1**^	**Self-reported model**^**1**^	**Physician-measured model**^**1**^
**Discrimination**			
AUC (95% CI)	0.67 (0.63, 0.72)	0.78 (0.75, 0.82)	0.83 (0.80, 0.86)
Change in AUC from the base model		0.108 (*P* <0.001)	0.152 (*P* <0.001)
Discrimination slope	0.099	0.242	0.306
Integrated discrimination index (IDI)		0.143	0.207
Sensitivity, given a specificity of 90%	23%	40%	53%
**Reclassification (compared to the base model)**			
*NRI (95% CI) based on quartile categories*			
Improvement in sensitivity		0.20 (0.13, 0.27)	0.20 (0.12, 0.27)
Improvement in specificity		0.17 (0.08, 0.26)	0.33 (0.24, 0.42)
Overall improvement in classification		0.37 (0.25, 0.48)	0.53 (0.41, 0.64)
*Category-free NRI*			
Improvement in sensitivity		0.33 (0.23, 0.42)	0.32 (0.23, 0.42)
Improvement in specificity		0.30 (0.18, 0.42)	0.53 (0.41, 0.65)
Overall improvement in classification		0.63 (0.47, 0.78)	0.85 (0.70, 1.01)
**Calibration**			
Hosmer-Lemeshow test *P* value	0.68	0.72	0.01
**Overall performance**			
Nagelkerke’s R^2^	0.131	0.315	0.393
Brier score	0.214	0.180	0.164
**Internal validation**			
Nagelkerke’s R^2^ (95% CI)	0.14 (0.09, 0.19)	0.34 (0.28, 0.41)	0.41 (0.35, 0.47)
AUC (95% CI)	0.68 (0.64, 0.71)	0.79 (0.76, 0.83)	0.83 (0.81, 0.86)
**External validation using data from English study**			
AUC (95% CI)	0.61 (0.57, 0.64)	0.71 (0.68, 0.74)	0.79 (0.76, 0.81)
Change in AUC from the base model		0.105	0.182
Discrimination slope	0.035	0.126	0.219
NRI (95% CI) based on quartile categories			
Improvement in sensitivity		0.20 (0.14, 0.25)	0.12 (0.07, 0.18)
Improvement in specificity		0.11 (0.04, 0.18)	0.34 (0.27, 0.41)
Overall improvement in classification		0.30 (0.22, 0.39)	0.46 (0.37, 0.55)
Hosmer-Lemeshow test *P* value	0.17	0.39	0.0003
Nagelkerke’s R^2^	0.050	0.166	0.303
Brier score	0.220	0.199	0.176

*AUC* Area under the receiver operating characteristic curve, *NRI* Net reclassification improvement.

^1^ The base model included demographic factors age, sex, city of recruitment and European ancestry. Both the self-reported model and the physician-measured model included the variables from the base model plus *MC1R*, non-melanoma skin cancer and nevi (categorised as ‘none, few some, many’ in the self-reported model, and as a continuous variable ‘the number of nevi ≥ 2 mm’ in the physician-measured model.

Compared to the base model, the self-reported model improved classification for a net 37% (95% CI 25-48%) of participants based on quartile cut-points and 63% (95% CI 47-78%) using the category-free approach; for the physician-measured model, net reclassification improvement was 53% (95% CI 41-64%) and 85% (95% CI 70-101%), respectively. Overall model performance also improved: Nagelkerke’s R^2^ increased from 13% in the base model to 32% for the self-reported model and 39% for the physician-measured model, and the Brier score decreased. Internal validation produced similar results for Nagelkerke’s R^2^ and the AUC. The discrimination slopes for each model (presented as box plots in Additional file 1: Figure S1), show how the physician-measured model achieved the best separation of predicted probabilities between cases and controls.

External validation of the final regression models using data from the English study showed slightly lower discrimination for the self-reported and physician-measured models compared to our Australian study results. However, this appeared to be due to lower discrimination for the baseline model (AUC 0.61 compared to 0.67), as both studies demonstrated similar improvements to the AUC, NRI and Nagelkerke’s R^2^ for the self-reported and physician-measured models when compared to the respective base model (Table 4). For both studies, the Hosmer-Lemeshow test indicated poor calibration (*P* <0.05) for the physician-measured model.

**Figure 1:**
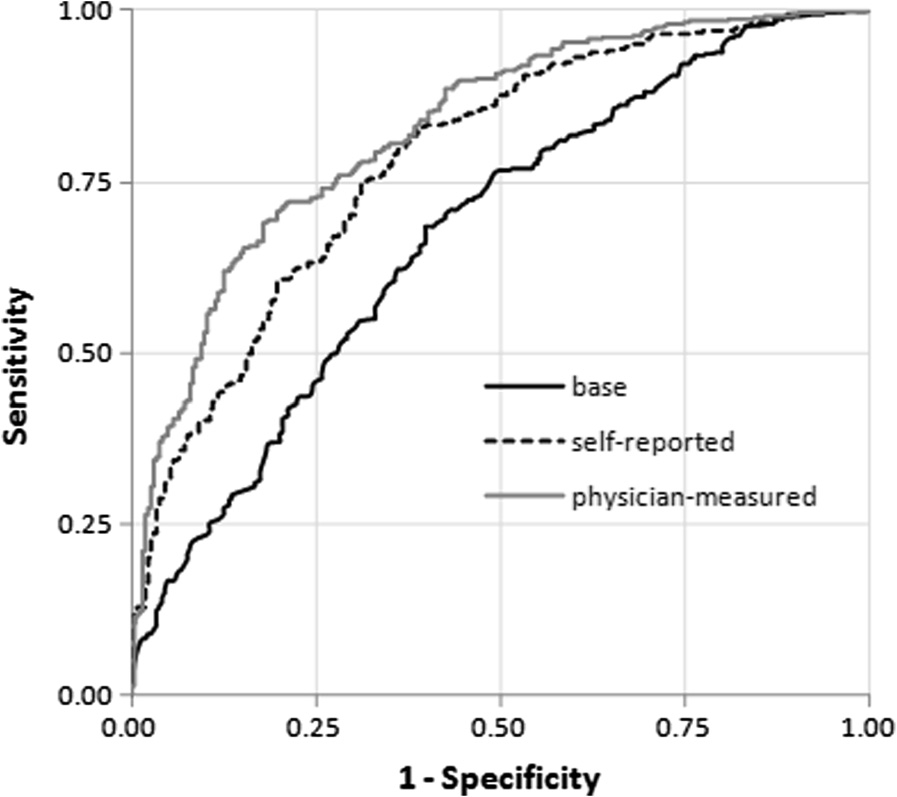
Receiver operating characteristic (ROC) curves for the base, self-reported and physician-measured final models.

Using ROC curves (Figure 1), we estimated the proportion of cases and controls that would be classified as high-risk using different cut-points. Choosing a cut-point value of 90% specificity, equating to 10% of controls being classified as high-risk, the proportion of cases identified as high-risk (i.e. sensitivity) was 23% in the base model, 40% in the self-reported model, and 53% in the physician-measured model. When we chose a different cut-point based on a balance of sensitivity and specificity (by selecting the value in the top, left-hand corner of the ROC plot), sensitivity and specificity were each 62% in the base model, 70% in the self-reported model and 74% in the physician-measured model.

### OR estimates

OR estimates for *MC1R*, nevi and non-melanoma skin cancer are presented in Table 5. Previously, we observed a stronger association between *MC1R* and melanoma for men than women in this study (*P* = 0.005) [15]; in this analysis the multivariate OR for any R allele compared to wild-type consensus alleles was 4.20 (95% CI 1.75-10.10) for men and 2.44 (95% CI 1.31-4.56) for women.

**Table 5:** **Odds ratios for melanoma associated with *MC1R*, nevi and non-melanoma skin cancer**

**Predictor**	**Cases**	**Controls**	**Minimally-adjusted odds ratio**^**1**^**(95% CI)**	**Multivariate-adjusted odds ratio**^**2**^**(95% CI)**
*MC1R*^3^				
Wild-type consensus alleles only	62	76	1.00	1.00
r only alleles	112	83	1.86 (1.16, 2.96)	1.72 (1.02, 2.89)
Any R allele	239	104	2.91 (1.88, 4.50)	2.94 (1.80, 4.80)
Number of physician-measured nevi ≥ 2 mm^4^				
Per 10 nevi increase	413	263	1.08 (1.06, 1.09)	1.08 (1.06, 1.10)
Self-reported nevi categories				
None	19	18	1.00	1.00
Few	113	135	0.68 (0.33, 1.42)	0.70 (0.32, 1.50)
Some	184	82	1.78 (0.85, 3.71)	2.01 (0.93, 4.36)
Many	97	28	2.71 (1.21, 6.10)	2.87 (1.23, 6.70)
Previous non-melanoma skin cancer				
No	382	259	1.00	1.00
Yes	31	4	8.84 (2.83, 27.63)	8.59 (2.68, 27.47)

^1^ Adjusted for age, sex, city of recruitment, and European ancestry.

^2^ Adjusted for age, sex, city of recruitment, European ancestry and other variables in the table (except nevi variables not adjusted for each other).

^3^ The categories are mutually exclusive. Silent changes (i.e. changes that are synonymous or occur in non-coding regions) are counted as consensus alleles.

^4^ Counted by a dermatology trainee.

### Incremental contribution of *MC1R* genotype to a model including traditional pigmentation variables, nevi and non-melanoma skin cancer

We assessed whether *MC1R* further improved prediction of melanoma when traditional pigmentation characteristics were already in the model, by replacing *MC1R* with ‘pigmentation score’ in the final regression models and then testing the addition of *MC1R.* We found that *MC1R* increased the AUC by 2.1% (from 0.77 to 0.79, *P* = 0.01) for the self-reported model and 1.3% (from 0.82 to 0.83, *P* = 0.04) for the physician-measured model; and improved the quartile classification for a net 10% (95% CI 1-18%, *P* = 0.03) of participants for the self-reported model and 6% (95% CI −1-13%, *P* = 0.08) for the physician-measured model (data not shown in tables).

## Discussion

*MC1R*, nevi and personal history of non-melanoma skin cancer were identified as the strongest predictors of melanoma risk in our study of early-onset melanoma. The contribution of *MC1R* to prediction of melanoma was similar to that obtained from measuring self-reported nevi, which is considered a strong and discriminative risk factor [5,33,34]. When added separately to the base model, *MC1R* and self-reported nevi increased the AUC by 6% and 5% respectively, and both improved classification for about a quarter of the cases and controls through net movement of cases into higher quartiles and controls into lower quartiles of predicted risk. Total number of nevi measured by physicians (dermatology trainees) was the strongest predictor of risk overall, increasing the AUC by 11% and reclassifying 39% of participants. Although *MC1R* genotype is strongly associated with skin and hair phenotype [12,14], it was a better predictor of early-onset melanoma than was pigmentation characteristics.

Our models demonstrated high discrimination: an AUC of 0.78 for the self-reported model and 0.83 for the physician-measured model in the Australian (development) dataset and 0.71 and 0.79 in the English (validation) dataset. The additional predictive value of *MC1R*, nevi and non-melanoma skin cancer variables when added to the demographic ‘base’ model was similar for both studies, suggesting good generalisability of our results to other genetically similar populations. The differences in the AUC for the base models of the two studies is less important, as it is strongly influenced by how age, sex, ethnic and regional differences have already been accounted for in the study design. It is expected that models will perform better on the development dataset than the validation dataset because of overfitting.

Three other, preliminary, melanoma risk prediction models containing *MC1R* genotype have been published. Whiteman and Green [16] published a prototype for a melanoma risk prediction tool but provided no details on predictive performance. In a published conference abstract, Smith *et al.*[17] showed an AUC of 0.72 (95% CI 0.70-0.75) for a model containing conventional risk factors and 0.75 (95% CI 0.72-0.77) when they added *MC1R* genotype, outdoor UV and indoor UV exposure, based on data from a case–control study of people aged 25–59 years in Minnesota. In a Greek study with 284 cases and 284 controls, Stefanaki and colleagues [18] derived a melanoma risk prediction model containing phenotypic traits (except nevi was not available) and 8 single nucleotide polymorphisms (SNPs) from several genes that included the *MC1R* locus. They found no appreciable change to the AUC after the addition of the 8 SNPs (AUC changed from 0.833 to 0.839), which they suggested may have been partly due to lower risk allele frequencies in their Greek population compared to other European populations [18]. Measurement of single SNPs rather than the causal variants might also underestimate the contribution of genetic variation to melanoma risk.

Other published risk prediction models for melanoma have reported AUCs in the range of 0.54 to 0.86 [34-41]. The highest reported AUC of 0.86 was for a model containing age, hair colour, personal history of melanoma and suspicious melanocytic lesion on dermoscopy, developed using a German cohort [36]. We were not able to include personal history of melanoma in our models because the study eligibility criteria specifically excluded these cases; however previous primary melanoma would be rare in people younger than 40 years and thus would be less relevant to our analysis than in studies with older participants. Previous non-melanoma skin cancer was a very strong risk factor in our study (OR 9), however because it had a low prevalence, the contribution to the AUC and more particularly to NRI was modest. This can be a limitation of these prediction methods, as factors that increase risk substantially can have minimal overall improvement to the model if they are rare. Another risk factor affected in this way is family history of melanoma. This variable did not improve discrimination in our analysis. We used a relatively low threshold for defining positive family history, requiring only one confirmed melanoma in a first-degree relative. This definition is consistent with many other population-based studies [7] and is associated with approximately two-fold increased melanoma risk [7,19]. However, a more extensive family history is associated with higher risk estimates [42] and thus, for some individuals, this factor will strongly influence their personal melanoma risk. This issue demonstrates the different priorities that are placed on the design of risk prediction tools for different settings: one type for stratifying the population into broad risk categories to aid primary prevention strategies, and the other type for more precisely estimating personal risk of melanoma.

Harbauer *et al.*[38] developed a model containing number of nevi, skin phototype and skin UV damage, adjusted for age and sex, with an AUC of 0.73 (95% CI 0.68-0.77) when measured by self-report and 0.77 (95% CI 0.73-0.83) when measured by a dermatologist. Similarly, our physician-measured model also achieved better discrimination than our self-report model, which was due solely to improved measurement of nevi. Our results indicate that counting all nevi greater than 2 mm is a better predictor of melanoma than counting only large, dysplastic or raised nevi. There was no added benefit to having physicians measure hair colour, eye colour or skin colour, as the discriminative ability of the pigmentation score was similar whether measured by self-report or by a physician.

Including sun and sunbed exposure variables resulted in little improvement to discrimination; when added as a group to the base model, they increased the AUC by 1.5%, but this diminished when the other factors were in the model. Although these sun and sunbed exposures have been shown to be associated with melanoma in our study [21,43], it has been demonstrated that very strong, independent associations with risk are required to meaningfully increase the AUC [29,44,45]. Sun exposure-related factors generally do not have very strong effect estimates, which may be partly due to inherent difficulties measuring past sun exposure [6] but also reflects the strong influence of genetically determined risk factors.

The Hosmer-Lemeshow test of calibration indicated poor agreement between observed and predicted outcomes for the physician-measured model, for both the development and external validation datasets. We examined the observed and expected values within each decile from the Hosmer-Lemeshow test but there no was consistent pattern describing the differences. Calibration has been rarely reported for other published melanoma risk prediction models. A prospective cohort study with follow-up of individuals for development of melanoma would be the ideal method to evaluate melanoma prediction probabilities.

Several strengths and limitations of the Australian Melanoma Family Study have been discussed previously [15,19,21]. We had low participation from cases and population controls, which is a common problem for population-based studies [46], especially when conducted with highly mobile, young adults [47]. Although poor participation can sometimes lead to selection bias [46], we did not find strong evidence of this occurring in our study [19]. This analysis was restricted to participants who had a complete set of data for self-reported and clinically-measured risk factors. Having a clinical skin examination was a preferred but not compulsory aspect of participation in the Australian Melanoma Family Study, and thus was only completed by a subset of case and control participants. Although this reduced the sample size for our analysis, it is unlikely to have introduced systematic bias, as a comparison of those with (n = 676) versus without (n = 270) clinical skin examinations showed no statistically significant differences on predictors including sex, self-reported nevi, hair colour, *MC1R* genotype, previous non-melanoma skin cancer, and childhood blistering sunburns.

Our study focused on prediction of early-onset melanoma. A younger age at diagnosis is more likely to reflect an underlying genetic susceptibility to the disease, thus the contribution of *MC1R* and traditional factors to risk prediction in our study may differ for those diagnosed at older ages or for other ethnicities. Nevertheless, our external validation results do suggest generalisability of our results to a broader-aged population. The generalisability of our results may also differ by country and the phenotypic and behavioural characteristics of the population. External validation of risk prediction models using an independent dataset has been rarely performed by other studies, and is an important strength of our study.

## Conclusions

Physician-measured nevi, *MC1R* genotype and previous non-melanoma skin cancer were the strongest predictors of early-onset melanoma in this study. Our results suggest that there might be modest benefit to measuring *MC1R* genotype for risk prediction even if information about traditional self-reported or clinically measured pigmentation characteristics and nevi is already available. Although many nonsynonymous *MC1R* gene variants exist [10,48], the six ‘R’ variants were responsible for most of the improvement to risk prediction in this study and thus would be the most important *MC1R* gene variants to include in melanoma prediction tools if it was not feasible or economically justifiable to measure all variants. We had limited statistical power to evaluate the predictive effect of individual R variants, however both our previous study [15] and a meta-analysis [14] attributed the R151C variant with the highest population burden based on prevalence and relative risk of melanoma.

Our study results will help guide the development of melanoma risk prediction tools that incorporate *MC1R* genotype. Decreasing genotyping costs and increasing use of genetic testing is making it more feasible to incorporate genetic risk factors into clinical risk prediction tools; however, translation into routine clinical practice requires several additional steps [49,50]. As a screening tool, evidence is needed to show whether or not individuals identified at high risk of melanoma will improve their sun-protection behaviours and perform regular skin checks, that improved outcomes justify the associated costs, and that the benefits of obtaining this information outweigh any disadvantages for patients and their families. For clinical genetic counselling, information on common variants in other melanoma susceptibility genes may need to be incorporated in the risk prediction tool in order to more precisely estimate personal risk of melanoma. Ultimately, the clinical and public health value of such tools will depend not only on its predictive performance, but also on the application setting, feasibility, cost effectiveness, benefits and harms [51,52].

## Abbreviations

MC1R: Melanocortin-1-receptor gene; ROC: Receiver operating characteristic curve; NRI: Net reclassification improvement; AUC: Area under the ROC curve; IDI: Integrated discrimination index; SNP: Single nucleotide polymorphisms.

## Competing interests

The authors declare that they have no competing interests.

## Authors’ contributions

AEC conceived the study idea, participated in its design, coordinated the statistical analysis, and drafted the manuscript. CG conducted the statistical analysis. MAJ and KV participated in the design of the study and helped to draft the manuscript. JRD and JHB conducted the statistical analysis for the external validation. EAH and CA participated in the *MC1R* sequencing. HS, BKA, RFK, JFA, GGG, DTB, JAN, JLH, and GJM coordinated the acquisition of data. All authors revised the manuscript critically for important intellectual content, and read and approved the final manuscript.

## Pre-publication history

The pre-publication history for this paper can be accessed here:

http://www.biomedcentral.com/1471-2407/13/406/prepub

## Supplementary Material

Additional file 1: Figure S1 Box-and-whisker plots showing the predicted probabilities of melanoma for cases and controls for the **A**. Base, **B**. Self-reported and **C**. Physician-measured final models. The discrimination slope is calculated as the difference between the mean predicted probability of melanoma for cases and controls. The box represents the median and interquartile range, and the bars indicate the range. The base model includes demographic factors age, sex, city of recruitment and European ancestry. Both the self-reported model and the physician-measured model also include *MC1R*, non-melanoma skin cancer and nevi (self-reported model = none, few, some, many; physician-measured model = number of nevi ≥ 2 mm). Click here for file
