# Supplementary figures and images for: MC1R genotype as a predictor of early-onset melanoma, compared with self-reported and physician-measured traditional risk factors: an Australian case-control-family study
Source: BMC Cancer. 2013 Sep 4;13:406. doi: 10.1186/1471-2407-13-406 (PMC3766240; doi:10.1186/1471-2407-13-406)

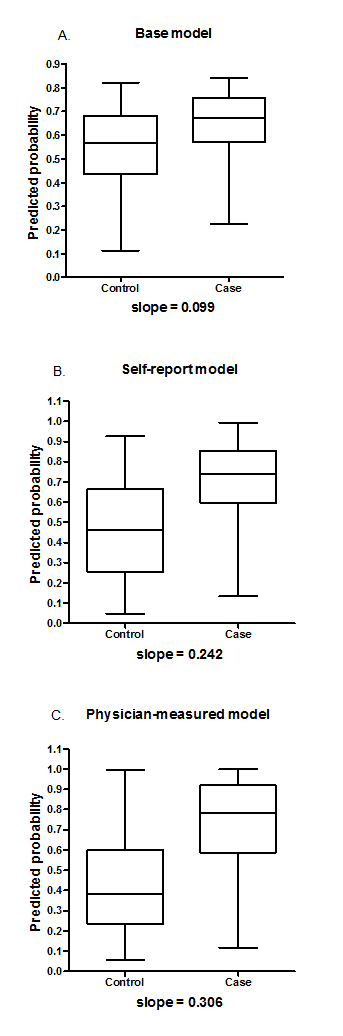

Supplement: Additional file 1: Figure S1 — Box-and-whisker plots showing the predicted probabilities of melanoma for cases and controls for the A. Base, B. Self-reported and C. Physician-measured final models. The discrimination slope is calculated as the difference between the mean predicted probability of melanoma for cases and controls. The box represents the median and interquartile range, and the bars indicate the range. The base model includes demographic factors age, sex, city of recruitment and European ancestry. Both the self-reported model and the physician-measured model also include MC1R, non-melanoma skin cancer and nevi (self-reported model = none, few, some, many; physician-measured model = number of nevi ≥ 2 mm). [file 1471-2407-13-406-S1.png]
